# Effect of whitening toothpastes on the surface roughness and microhardness of human teeth—an in vitro study

**DOI:** 10.1007/s00784-023-05381-9

**Published:** 2023-11-15

**Authors:** Navodita Jamwal, Ashwini Rao, Gowri Shankar MC, Ramya Shenoy K, Mithun Pai BH, Praveen Jodalli, Aparna KS, Avinash BR

**Affiliations:** 1 Public Health Dentistry, Manipal College of Dental Sciences, Mangalore, Manipal Academy of Higher Education, Manipal, Karnataka 576 104 India; 2 Mechanical and Industrial Engineering, Manipal Institute of Technology, Manipal Academy of Higher Education, Manipal, 576104, India

**Keywords:** Dental enamel, In vitro study, Microhardness, Surface roughness, Whitening toothpaste

## Abstract

**Objective:**

To determine the effect of whitening toothpastes on the surface roughness and microhardness of human teeth.

**Methodology:**

Surface roughness was estimated using the Talysurf instrument, and microhardness was estimated using the Vickers hardness tester before and after the application of whitening toothpastes on mounted extracted human teeth.

**Results:**

In the activated charcoal group, there was a reduction in the surface roughness from 1.21 at baseline to 1.09 at 1 month and a further reduction to 1.02 at 3 months, which was found to be statistically significant. However, no statistically significant difference in surface roughness was found in the other toothpaste groups. With respect to microhardness, all 4 whitening toothpastes showed a statistically significant reduction in microhardness after 3 months of brushing. However, the reduction was significantly higher in group 2 and in group 4 compared to the others.

**Conclusion:**

This study showed that whitening toothpaste containing activated charcoal significantly reduced the surface roughness, whereas toothpastes with blue covarine and toothpastes containing activated charcoal significantly reduced the microhardness of the tooth.

**Clinical relevance:**

This study emphasizes the need for healthcare professionals to be aware of the potential disadvantages of whitening toothpastes and make evidence-based decisions when recommending the product to patients.

## Introduction

People have been fascinated by pearly white teeth since times immemorial. Studies in both India [1] and other world populations [2–4] have shown that 20–65% of people are dissatisfied with their tooth color.

Tooth color is affected by the inherent color of the teeth and/or any extrinsic stains that may develop on the tooth surface [5]. Traditionally, tooth discoloration is classified as intrinsic or extrinsic. Intrinsic stains are present inside the tooth and are caused by agents introduced during the formation of teeth [6]. Extrinsic staining is related to the components that bind to the acquired pellicle on the surface of enamel, resulting in staining of the teeth. Poor tooth brushing technique, smoking, consumption of colored food, age and the use of specific cationic agents such as chlorhexidine or metal salts such as tin and iron are among the factors that may cause extrinsic staining of teeth [6].

Scaling, polishing, bleaching, and the use of prosthetic crowns are all professional ways to whiten teeth. However, individuals can also whiten their own teeth at home with over-the-counter (OTC) whitening toothpastes [7]. During professional tooth whitening, structural damage to enamel surface prisms and increased tooth sensitivity have been documented in many studies [8–10].

The whitening components used in toothpastes can be abrasive agents such as hydrated silica, calcium carbonate, dicalcium phosphate dehydrate, calcium pyrophosphate, alumina, perlite, or sodium bicarbonate. They could also be chemical agents such as hydrogen peroxide, calcium peroxide, sodium citrate, sodium pyrophosphate, sodium tripolyphosphate, sodium hexametaphosphate, or papain. They could also be optical agents, such as blue covarine [11]. Because activated charcoal has the ability to absorb pigments and stains, certain toothpastes are now incorporating it as a whitening agent [7]. Abrasives are included in all toothpastes; however, whitening toothpastes frequently have a higher concentration of harsher abrasives [12].

Studies have shown that the clinical efficacy of whitening toothpastes has been contradictory, with some demonstrating an improvement in tooth color [13, 14] and others finding very little clinically relevant effect on tooth whitening [15–17]. Casado et al. [18], in their systemic review on the efficacy of dental bleaching, suggested that despite the evidence suggesting that whitening toothpastes are effective in improving tooth color, it is important to examine their effect on the tooth surface. Benahmed et al. [19], in their review on natural teeth whitening, reported that natural teeth whiteners lighten the color of teeth without eroding the tooth surface. However, they also reported that commercially available whiteners could lead to deproteination and demineralization of teeth in higher concentrations when used extensively. Schwarzbold et al. reported that toothpastes that claim to be whitening merely have an abrasive impact, and they do not actually bleach the tooth structures [20]. Studies [21–26] have shown that whitening toothpastes affect the surface roughness and hardness of enamel.

Jamwal et al. [27], in their systematic review and meta-analysis, reported that although these toothpastes brought about some amount of tooth lightening, these toothpastes also affected the mineral content of teeth by increasing surface roughness and reducing microhardness. They also suggested the need for further research into the effect of the particular whitening agent, which would not only whiten the tooth effectively but also maintain the integrity of the tooth structure. The paucity of studies in this area fostered a need for this study. Inadequate evidence about the deleterious effect of these whitening agents on the tooth surface may lead to a cavalier attitude during the use of these toothpastes, resulting in devastating consequences. This was the rationale of this study, with the aim of determining the effect of whitening toothpastes marketed in India on the surface roughness and microhardness of teeth. The objective was to identify their effect on human teeth based on the type of whitening agent used. The research hypotheses for this study were the following: H1—the surface roughness and microhardness of the teeth brushed with whitening toothpastes will be similar to that of the teeth brushed with a nonwhitening toothpaste; and H2—the surface roughness and microhardness of the teeth brushed with whitening toothpastes will show changes when compared to that of the teeth brushed with a nonwhitening toothpaste.

## Methodology

### Study design

This study was a randomized, controlled, double-blinded in vitro study.

### Sample size determination

Sample size was calculated using G*Power software (version 17 March 2020 – Release 3.1.9.7, Heinrich-Heine Dusseldorf University, Dusseldorf, Germany), based on 80% power, 95% confidence interval, with effect size of 0.5, was determined to be 5 samples in each group. The reference values were taken from the key article [24].

### Tooth collection and preparation

**Fig. 1:**
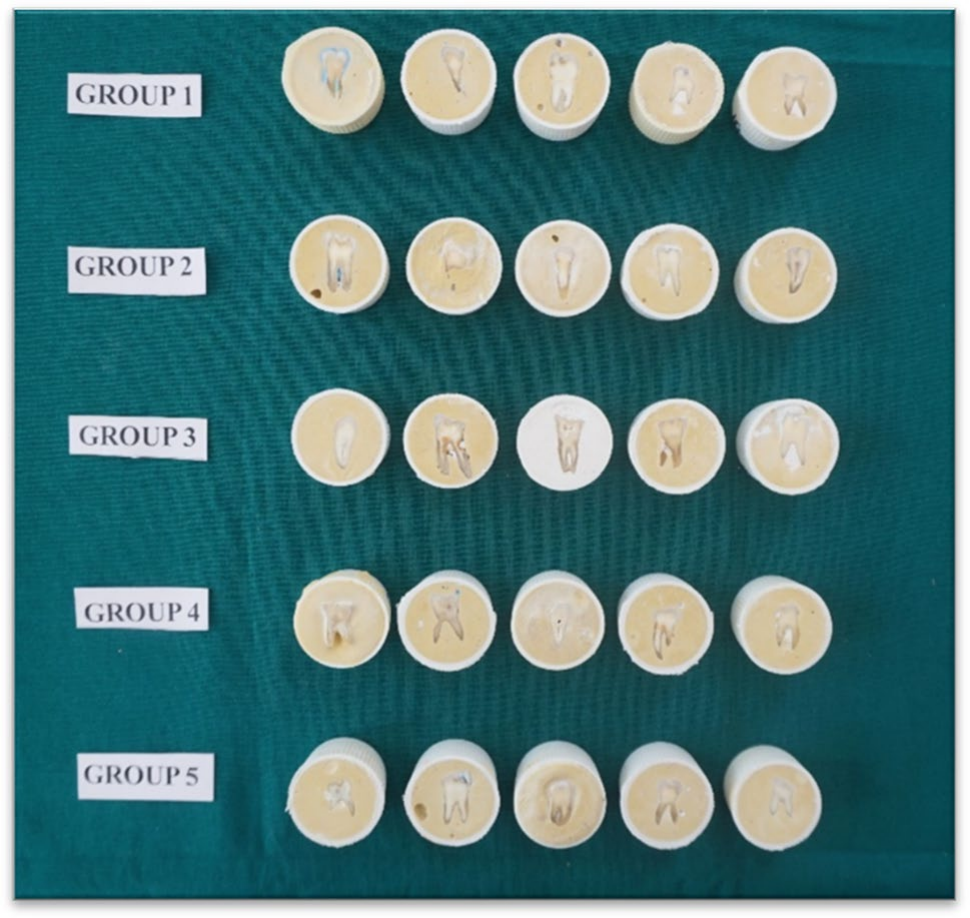
Mounted teeth

Freshly extracted human teeth were collected from outpatient departments. The teeth were inspected to exclude those with dental caries, restorations, fractures, attrition or abrasion. The selected teeth were mounted in gypsum plaster, exposing the buccal surface of the tooth. Twenty-five extracted sound human teeth were thus prepared (Fig. 1).

### Toothpaste groups

There were 5 toothpaste groups. Group 1 to group 4 were the whitening toothpastes, and group 5 was the control. Group 1 included Pepsodent whitening germicheck toothpaste, whose active ingredient was perlite. Perlite is an abrasive agent that is a chemically inert, amorphous, glassy silicate of volcanic origin with a neutral pH. It is one of the ingredients used in dental prophylaxis paste used for polishing teeth. It has been shown to enhance the stain removal properties of silica-based toothpaste [28].

Closeup diamond attraction whitening toothpaste belonged to group 2, whose active ingredient was blue covarine, which is an optical agent causing deposition of a thin, semitransparent layer of blue dye pigment on the enamel surface. The interaction between incident light and this film changes the color of the reflected teeth from the yellow region to the blue region, giving the impression that they are whiter and brighter [17].

Group 3 consisted of Colgate Visible White Plus Shine toothpaste, whose active ingredient was sodium tripolyphosphate. Chemically speaking, sodium tripolyphosphate (STP) is a linear condensed phosphate used in whitening toothpastes. Condensed phosphates are polyanionic, surface-active compounds that have been added into toothpastes due to their ability to prevent mineralization. Additionally, they have the ability to desorb salivary proteins from enamel, prevent protein adsorption to these substrates and perhaps prevent and lessen tooth staining [29].

Colgate Charcoal Clean toothpaste was categorized as group 4 with activated charcoal as the active ingredient. Activated charcoal is a fine powder with variable abrasivity obtained by oxidizing carbon-rich material through controlled reheating or chemical means. It has been suggested that activated charcoal binds to deposits on teeth, which are then brushed away and leave tooth surfaces free of any deposits [30].

Group 5 was the control consisting of Pepsodent 2 in 1 toothpaste, which was a nonwhitening toothpaste.

### Brushing procedure

Brushing teeth for the necessary 120 s twice daily results in a total of 56 locations with buccal and lingual/palatal surfaces if an adult has at least 28 teeth. Thus, spending 5 s on each tooth per day, the samples were brushed for a total of 150 and 450 s to simulate one month and three months, respectively [25], using a pea-sized measure of the toothpaste squeezed against the bristles.

### Blinding

All tooth specimens were numbered and randomly allocated into the five groups using a list of random numbers created by RANDOM.ORG [31]. All toothpastes were covered to conceal their identity by one of the investigators (AR), and the principal investigator (NJ) was also blinded to the group to which the tooth belonged.

### Surface roughness measurement

**Fig. 2:**
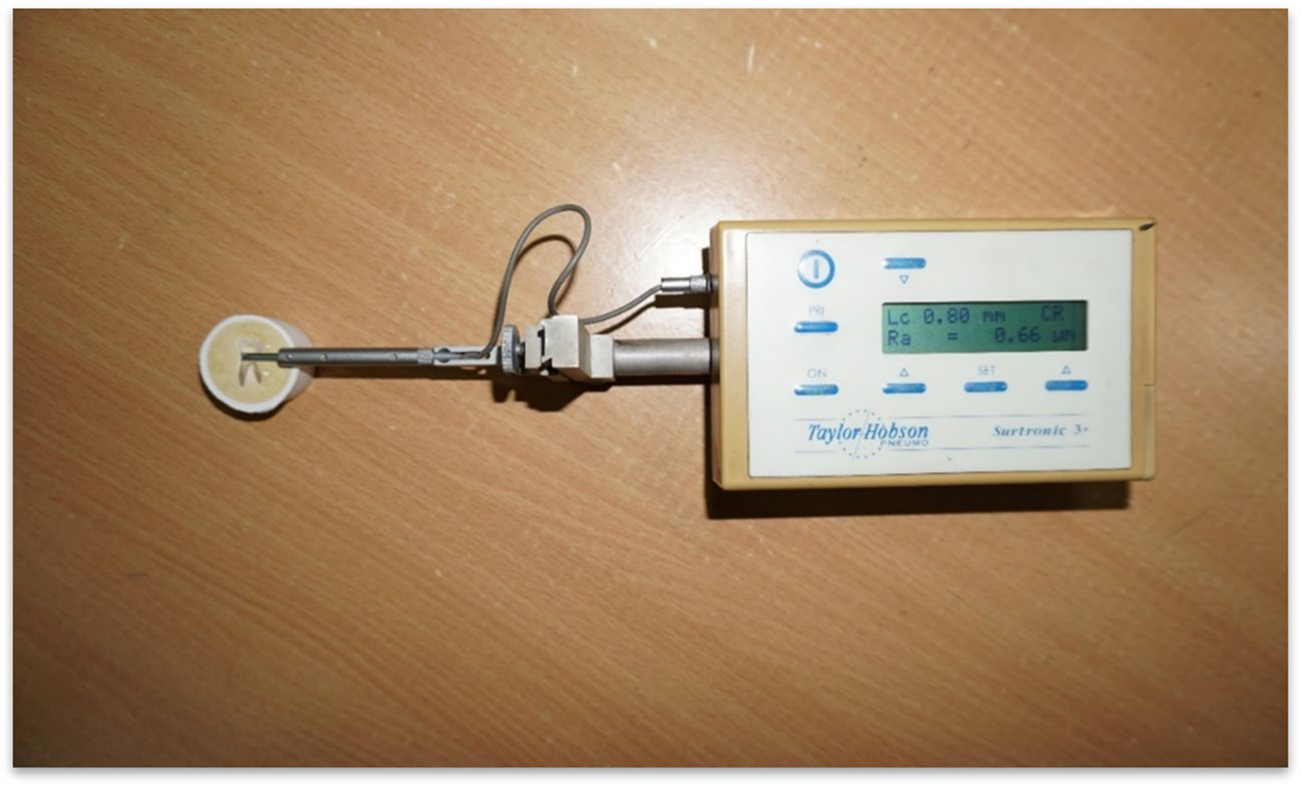
Surface roughness testing in progress

The surface roughness of all specimens was measured using the Taylor and Hobson Surface Roughness Tester (Talysurf Instrument) (Fig. 2). The surface roughness instrument has a stylus tip of a radius of 2 μm with a cut-off value of 0.8 mm and was used for a surface data length of 5 mm. The central region of each tooth specimen was chosen for measurement. The stylus diamond tip had to be moved vertically over the tooth surface [32]. The surface roughness was recorded in Ra before and after brushing procedures. Each specimen was recorded three times at each step (i.e., for baseline, 1 month, and 3 months) at different locations of the tooth surfaces for a total of three successive randomized readings that were then converted into a mean value [25].

### Microhardness (VHN) measurement

**Fig. 3:**
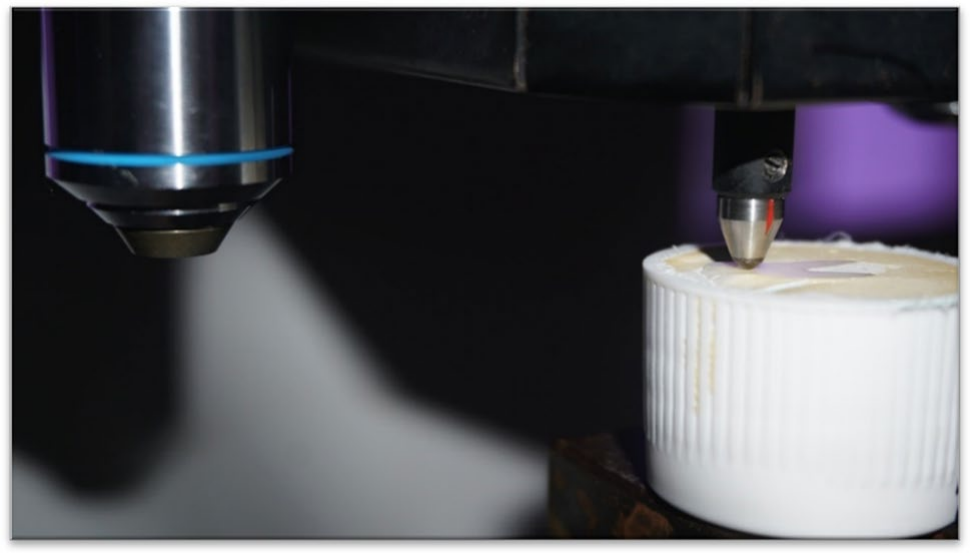
Microhardness testing in progress

**Fig. 4:**
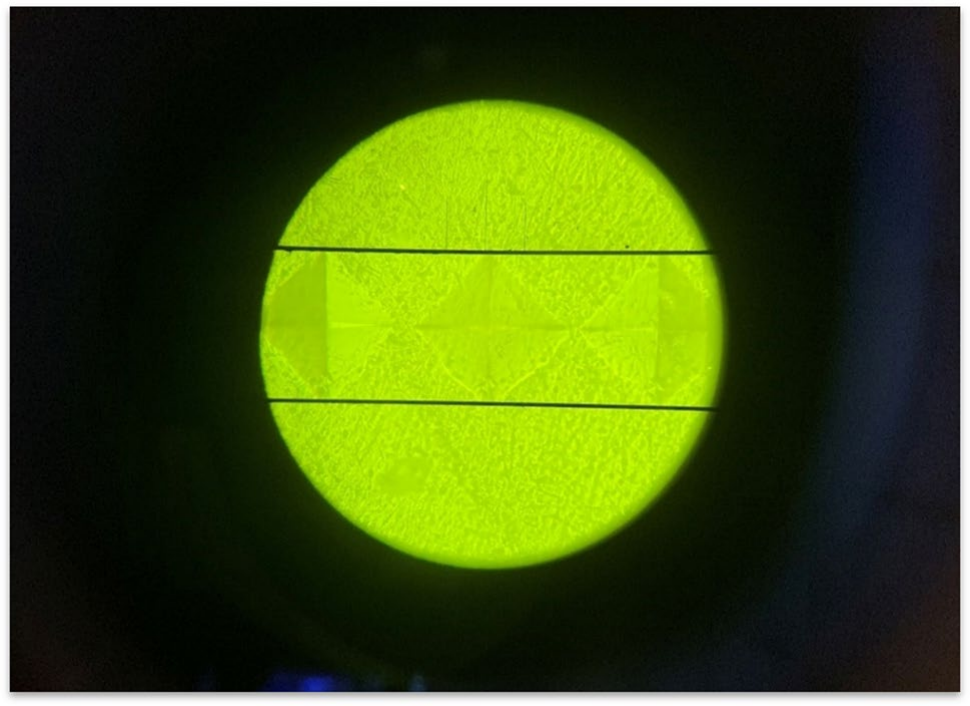
Diamond-shaped indentations on the tooth surface

The Vickers hardness number (VHN) of all specimens was measured using a Vickers hardness tester (Matsuzawa Co., Ltd., Akita Pref, Japan Matsusawa microhardness tester) (Fig. 3). Each tooth specimen was impressed with a load of 300 gm with a dwell time of 15 s. The hardness was recorded in VHN by making 3 indentations from the center of the specimen and 100 μm apart from each other (Fig. 4). The average of the 3 VHN recordings was then calculated [33].

## Statistical analysis

The data were coded and analyzed using SPSS version 11.5. Descriptive statistics were first obtained. To determine whether there was any statistically significant difference in the surface roughness or microhardness values at the 3 time periods, i.e., baseline, 1 month, and 3 months, within each group, a paired t-test was performed, applying the Bonferroni correction.

A repeated-measure analysis was then performed to determine the presence of any significant difference between the 5 groups. Then, the Tukey test was applied to determine which of the groups showed statistically significant differences.

## Results

This was an in vitro study conducted on extracted human teeth to determine the effect of whitening toothpastes on the surface roughness and microhardness of human teeth. The teeth were allotted into 5 groups, brushed with 4 whitening toothpastes with different kinds of whitening agents and one regular fluoridated toothpaste, which was the control (Table 1).

**Table 1:** Study groups and toothpaste contents

Groups	Product name	Ingredients	Tooth whitening technology used
Group 1	Pepsodent whitening germicheck	Calcium carbonate, water, sorbitol, hydrated silica, sodium lauryl sulphate, potassium nitrate, flavour, sodium monofluorophosphate, perlite, cellulose gum, sodium silicate, benzyl alcohol, sodium saccharin, CI 74160, CI 74260, CI 77891	Perlite (abrasive)
Group 2	Closeup diamond Attraction Whitening	Sorbitol, water, hydrated silica, sodium lauryl sulphate PEG-32, flavour, cellulose g, trisodium phosphate, sodium fluoride, sodium saccharin, PVM/MA copolymer, C174160, Mica/Cl77019, limonene, CI 77891	Blue covarine (optical pigment)
Group 3	Colgate Visible White Plus Shine	Silica, sorbitol, glycerine, polyethylene glycol, pyrophosphate, sodium tripolyphosphate, tetrapotassium pyrophosphate, sodium lauryl sulphate, flavour, cocamidopropyl betaine, sodium carboxymethyl cellulose, sodium monofluorophosphate, xanthan, sodium saccharin in aqueous base	Sodium tripolyphosphate (chemical)
Group 4	Colgate Charcoal Clean	Sorbitol, water, silica, sodium lauryl sulphate, flavour, cocamidopropyl betaine, polyethylene glycol 600, sodium carboxymethyl cellulose, sodium saccharin, sodium fluoride, charcoal, benzyl alcohol, eugenol	Activated charcoal (abrasive)
Group 5-control	Pepsodent 2 in 1	Water, sorbitol, calcium carbonate, hydrated silica, sodium lauryl sulphate, flavour, PEG-32, sodium monofluorophosphate, cellulose gum, trisodium phosphate, benzyl alcohol, sodium saccharin, CI 77891, CI 74160, limonene	Nonwhitening toothpaste

The surface roughness (Ra) and microhardness (VHN) of 25 extracted human tooth specimens were measured using the Talysurf Instrument and the Vickers hardness tester, respectively, at baseline and after 1 month and 3 months of brushing using 4 different whitening toothpastes and a nonwhitening toothpaste as a control. All toothbrushings were carried out using a motorized toothbrush with separate heads and toothpastes for each group.

### Surface roughness

Table 2 shows the mean surface roughness scores for the 5 groups at baseline (before brushing) and at 1 month and 3 months after brushing. In group 1 (perlite), the surface roughness was found to be 0.99 at baseline and reduced to 0.81 at 1 month but showed a slight increase at 3 months, although the difference was not statistically significant. In group 2 (blue covarine), the surface roughness values decreased from 1.21 at baseline to 1.15 at 1 month and remained the same at 3 months, but the difference was not found to be statistically significant. In group 3 (STP), there was a reduction in the surface roughness scores from 1.38 at baseline to 1.32 at 1 month and to 1.07 at 3 months. However, this was not found to be statistically significant. In group 4 (activated charcoal), there was a reduction in the surface roughness from 1.21 at baseline to 1.09 at 1 month and a further reduction to 1.02 at 3 months, which was found to be statistically significant. In group 5 (control), although the surface roughness showed a reduction from 1.27 at baseline to 1.13 at 1 month, the value increased to 1.21 at 3 months, although the difference was not statistically significant. However, when the surface roughness values were compared between the toothpaste groups, no statistically significant difference was found between the 5 toothpaste groups. (*F* = 2.259; *P* > 0.05) (Table 2).

**Table 2:** Surface roughness scores between and within groups

Group	*N*	Surface roughness Baseline	Surface roughness 1 month	Surface roughness 3 months	*P* value
1	5	0.99 ± 0.23	0.81 ± 0.29	0.89 ± 0.34	> 0.05
2	5	1.21 ± 0.51	1.15 ± 0.38	1.15 ± 0.24	> 0.05
3	5	1.38 ± 0.77	1.32 ± 0.51	1.07 ± 0.33	> 0.05
4	5	1.21 ± 0.40*	1.09 ± 0.65	1.02 ± 0.43*	= 0.014
5	5	1.27 ± 0.27	1.13 ± 0.55	1.21 ± 0.60	> 0.05

^*^Within groups *F* = 2.259; *P* > 0.05 paired *t*-test, applying the Bonferroni correction

### Microhardness

Table 3 shows the mean microhardness scores between groups at baseline (before brushing) and at 1 month and 3 months after brushing.

**Table 3:** Microhardness scores between and within groups

Group	N	Microhardness Baseline	Microhardness 1 month	Microhardness 3 months	*P* value
1	5	70.72 ± 9.15*	57.28 ± 6.66	53.35 ± 9.23*	= 0.000
2	5	61.85 ± 15.16*	54.97 ± 8.30	53.00 ± 7.30*	= 0.004
3	5	61.99 ± 7.21	66.06 ± 14.89*	57.23 ± 6.54*	= 0.017
4	5	62.04 ± 9.96*	52.38 ± 9.23	49.51 ± 4.77*	= 0.001
5	5	66.23 ± 8.02	63.79 ± 7.66	64.33 ± 13.69	> 0.05

^*^Within groups *F* = 4.875; *P* = 0.002 paired *t*-test, applying the Bonferroni correction

In group 1 (perlite), microhardness values showed a decreasing trend from 70.72 at baseline to 57.28 at 1 month and 53.35 at 3 months, and this reduction in microhardness was found to be statistically significant. In group 2 (blue covarine), there was a reduction in the microhardness value from 61.85 at baseline to 53.0 at 3 months, and this was found to be statistically significant. In group 3 (STP), the microhardness value was 61.99 at baseline and increased to 66.06 in the first month, and this was not found to be statistically significant. However, at the third month, the value drastically reduced to 57.23, which was found to be statistically significant compared to baseline. In group 4 (activated charcoal), there was a reduction in the microhardness value from 62.04 at baseline to 53.35 at 3 months, and this was found to be statistically significant. In group 5 (control), although there was a slight reduction in the microhardness value from 66.23 at baseline to 63.79 at 1 month and the value increased to 64.33 at 3 months, this was not found to be statistically significant. With respect to microhardness, all 4 whitening toothpastes showed a statistically significant reduction in microhardness after 3 months of brushing. When the microhardness values between the 5 toothpaste groups were compared, it was found that there was a statistically significant difference between the groups (*F* = 4.875; *P* = 0.002). To identify which of the groups showed a significant difference in the microhardness values, the Tukey test was applied and a statistically significant higher reduction in the microhardness value was found in group 2 and in group 4 compared to the other two groups (Table 4).

**Table 4:** Post hoc Tukey test for microhardness

Reference group	Comparison group	Mean difference	Std. error	Significance	95% confidence interval	
					Lower bound	Upper bound
1	2	3.8467	2.60057	0.579	− 3.4353	11.1286
	3	− 1.3067	2.60057	0.987	− 8.5886	5.9753
	4	5.8111	2.60057	0.179	− 1.4709	13.0931
	5	− 4.3444	2.60057	0.459	− 11.6264	2.9375
2	3	− 5.1533	2.60057	0.286	− 12.4353	2.1286
	4	1.9644	2.60057	0.942	− 5.3175	9.2464
	5	− 8.1911^*^	2.60057	0.020	− 15.4731	− .9091
3	4	7.1178	2.60057	0.059	− .1642	14.3998
	5	− 3.0378	2.60057	0.769	− 10.3198	4.2442
4	5	− 10.1556^*^	2.60057	0.002	− 17.4375	− 2.8736

^*^Based on observed means, the mean difference is significant at the.05 level (Values in bold indicate statistical significance)

## Discussion

This was an in vitro study conducted on extracted human teeth to highlight the effect of different whitening agents used in whitening toothpastes. Toothpastes have been routinely used for plaque removal and contain different abrasives, such as silica, calcium carbonate, calcium phosphate, and sodium bicarbonate, in addition to therapeutic agents [34]. Whitening toothpastes are known to have chemical agents or newer abrasives to intensify extrinsic stain removal and bring about tooth whitening. However, in addition to the beneficial effect of tooth whitening, some of these toothpastes have been shown to result in increased surface roughness and a reduction in the microhardness of teeth [23–25]. Based on the results, the second research hypothesis was accepted because the surface roughness and microhardness of the teeth brushed with whitening toothpastes showed changes when compared to that of the teeth brushed with a nonwhitening toothpaste.

### Surface roughness

When the surface roughness scores of each toothpaste group was compared after brushing for 1 month and 3 months from baseline, it was found that except for group 4, none of the other toothpaste groups showed any statistically significant change in surface roughness scores. However, teeth categorized under group 4 (brushed with toothpaste containing activated charcoal) showed a statistically significant reduction in surface roughness values from 1.21 at baseline to 1.02 at 3 months of brushing.

When each of the toothpaste groups was compared, no statistically significant difference was found between the 5 toothpastes with respect to surface roughness values at one and three months of toothbrushing. Therefore, this study showed that only one toothpaste, i.e., group 4—the activated charcoal group, reduced the surface roughness of teeth, whereas none of the other toothpastes had any impact on the surface roughness of teeth brushed in vitro. Similar results were obtained by Alpan and Özdede [25] in their in vitro study on human enamel, where they reported that surface roughness decreased significantly in two whitening toothpastes, group 3 and group 4, brushed for 5 s each day for one month. In group 3 (splat special blackwood), the active ingredient was charcoal powder, and in group 4 (Colgate optic white), although the active ingredient was hydrogen peroxide, it also had coal, which is known to have high abrasivity. However, Vural et al. [33] reported a substantial increase in surface roughness in enamel brushed for 12 weeks with charcoal-based whitening toothpastes, which is in contrast to the findings of the present study. They claimed that the increase in surface roughness could be influenced by the presence of one of the most common abrasives, silica, in these toothpastes. Similar findings were reported by Bolay et al. [22], where surface roughness was found to increase when brushed with natural white whitening toothpaste, which contained pentasodium triphosphate. Feitosa et al. [23] found that there was a statistically significant increase in the surface roughness values of enamel brushed with whitening toothpastes Colgate total advanced whitening and Colgate whitening oxygen bubbles. However, the control toothpaste, Colgate total advanced clean, also showed a statistically significant increase in the Ra value after 10 h of brushing. The authors concluded that all three of the toothpastes used in that study contained abrasives that could have promoted surface alterations in enamel. Maden et al. [35] reported that Ipana white power carbonate toothpaste showed a statistically significant increase in the surface roughness of teeth brushed for 2 min, twice a day, for a week, and Rahardjo et al. [24] also reported a significant increase in enamel roughness after 1 and 3 months of equivalent tooth brushing with 2 whitening toothpastes, one containing perlite and the other containing ε-phtalimido peroxycaproid acid. However, Shamel et al. [36] reported no statistically significant difference in the mean values of surface roughness of all groups used in their study (Closeup white now, Sensodyne true white, Colgate optic white). Although Jamwal et al. [27], in their meta-analysis, revealed that surface hardness showed an increase in teeth brushed with whitening toothpastes, they also stressed the need for further research to identify the type of whitening agent that would maintain the integrity of the tooth surface.

### Microhardness

When the microhardness values within the toothpaste groups were analyzed, it was found that groups 1, 2, 3, and 4, i.e., perlite, blue covarine, STP, and activated charcoal, showed a statistically significant reduction in the microhardness values at 3 months of brushing compared to baseline. However, the control group, group 5, did not show any changes in the microhardness values at 1 month and 3 months of brushing.

When different toothpastes were compared to determine which of the toothpastes brought about a higher reduction in microhardness in comparison to others, it was found that toothpastes of groups 2 (blue covarine) and 4 (activated charcoal) showed a higher reduction in microhardness values compared to the control toothpaste, and this difference was statistically significant. Although studies [7, 36] have shown that the best tooth whitening performance was obtained using microbeads and blue covarine, the present study showed that toothpastes using blue covarine and activated charcoal also reduced the microhardness of teeth in vitro. However, Joiner et al. [37], in their in vitro study on bovine enamel, showed no statistically significant effect on enamel when brushed with toothpaste containing blue covarine. Bolay et al. [22], in their in vitro study, did not find any statistically significant effect of the whitening agent pentasodium triphosphate toothpaste, present in natural white whitening toothpaste, on enamel hardness. Vural et al. [33] also did not find any change in the microhardness of enamel brushed for 12 weeks with charcoal-based whitening toothpastes, which is in contrast to the present study, where a significant reduction in microhardness was observed among teeth brushed with Colgate charcoal clean, which had active charcoal as the whitening agent. Greenwall et al. [30] suggested that given the high absorption capacity of activated charcoal, it is possible that any fluoride and other active ions present in charcoal-based toothpaste will not be available for remineralizing enamel, let alone boosting its resistance to caries and other processes that cause tooth attrition. Rahardjo et al. [24] reported a significant reduction in microhardness after 1 and 3 months of equivalent tooth brushing with 2 whitening toothpastes, one containing perlite and the other containing ε-phtalimido peroxycaproid acid. This was in contrast to the present study, where the perlite-containing toothpaste did not have any significant effect on the microhardness of the teeth brushed. Maden et al. [35] reported a statistically significant reduction in the enamel surface microhardness for Ipana White Power Carbonate Toothpaste after tooth brushing for 2 min, twice a day, for a week. Khamverdi et al. [21], in their study comparing the effect of whitening toothpaste on the microhardness of enamel and composite resin, found that crest whitening (special silica abrasives as the whitening component) and Aquafresh whitening toothpastes (sodium tripolyphosphate as the whitening component) did not affect enamel hardness but reduced the microhardness of composite resin.

Whitening toothpastes achieve their effect either by oxidizing with peroxide, which chemically alters dental proteins, by adding abrasive ingredients that remove stains or by applying colorants, such as covarine, to the surface of the tooth, giving the immediate impression of whiter teeth. When the primary means by which a toothpaste whitens teeth is through the abrasives it contains, an ideal balance between abrasiveness and whitening effect is a challenge to achieve. It is evident that the current situation calls for safer whitening solutions that significantly whiten teeth without compromising the roughness or hardness of the tooth surface.

## Limitations

Although all 4 whitening toothpastes showed a reduction in microhardness after 3 months of brushing, long-term studies reflecting the oral environment and adjusting for the effect of silica abrasives are needed to determine the effect of whitening toothpastes in general and the whitening component in particular on the surface roughness and microhardness of teeth. The composition of toothpastes with respect to the type, size, and quantity of abrasives was not clearly depicted on the labels, which made it difficult to identify the different types of other abrasives present in the formulation.

## Conclusion

Within the limitations, this study found the following:

1. Toothpastes containing activated charcoal significantly reduced the surface roughness of teeth in vitro.
2. Toothpastes with blue covarine and those containing activated charcoal significantly reduced the microhardness of teeth in vitro.

While sufficient evidence exists regarding the effectiveness of whitening toothpastes in improving tooth color, it was imperative to also look at the other side of the coin, where literature was limited. The focus of this study therefore was to find the effect of different whitening agents on the surface roughness and microhardness of teeth in vitro. Although four whitening toothpastes with different types of whitening agents were analyzed, the type and size of the abrasives in the toothpastes could not be identified which could act as a confounding factor. However, since blinding was done and the methodology used was robust, the conclusions can be generalized and it can be concluded that whitening toothpastes need to be used with caution.

## Clinical relevance

The production, promotion, and use of over-the-counter, at-home whitening toothpastes have multiplied, as public interest in tooth whitening has grown and clinicians frequently promote whitening toothpastes as a home cure for teeth whitening. This study emphasizes the need for healthcare professionals to be aware of the potential disadvantages of these products and make evidence-based decisions when recommending the product to patients.

## Data Availability

The datasets that support the findings of this study are not openly available but can be obtained from the corresponding author upon request.
